# Complete mitochondrion genome of the endangered Mohave tui chub *Siphateles bicolor mohavensis* (Cypriniformes: Cyprinidae)

**DOI:** 10.1080/23802359.2017.1398602

**Published:** 2017-11-08

**Authors:** Frances L. Glaser, Kylie S. Owen, Sujan M. Henkanaththegedara, Steve Parmenter, Craig A. Stockwell, Sean C. Lema

**Affiliations:** a Department of Biological Sciences, California Polytechnic State University, San Luis Obispo, CA, USA;; b Biological and Environmental Sciences, Longwood University, Farmville, VA, USA;; c California Department of Fish and Wildlife, Bishop, CA, USA;; d Department of Biological Sciences, North Dakota State University, Fargo, ND, USA

**Keywords:** mtDNA, mitochondria DNA, conservation, genetics, fish

## Abstract

The Mohave tui chub (*Siphateles bicolor mohavensis*) is an endangered cyprinid fish endemic to the Mojave Desert region of southeastern California, USA. Here, we describe the complete 16,607 base pair (bp) mitochondrial genome of *S. b. mohavensis*. The mitogenome has a nucleotide base composition of A (29.08%), T (26.91%), G (17.58%), and C (26.43%), and encodes 13 protein subunits, 22 tRNAs, a 12S rRNA of 956 bp and 16S rRNA of 1691 bp, and a 929 bp D-loop control region, each located in the conserved mtDNA structure typical for cyprinid fishes. All protein-coding genes have initiation codons of ATG or GTG, and only the *ND1*, *CO1*, *ATPase8*, *NDL4*, *ND4, ND5*, and *ND6* genes have complete stop codons. Phylogenetic analyses confirmed the relationship of *S. b. mohavensis* to several genera of cyprinids (e.g. *Gila*, *Acrocheilus*) also endemic to western North America. This characterized mitogenome may help inform management practices for *S. b. mohavensis* by facilitating future studies on how allopatric populations of this imperiled species are evolving across refuge habitats.

The Mohave tui chub *Siphateles bicolor mohavensis* (Snyder 1918; formerly *Gila bicolor mohavensis*) is a medium-sized (<40 cm) cyprinid that is the only fish endemic to the arid Mojave River drainage of California, USA (Hubbs and Miller 1943). *Siphateles bicolor mohavensis* was thought to be widespread throughout the Mojave River drainage during the Pleistocene (Buwalda 1914; Blackwater and Ellsworth 1936), but was abundant only in the river’s lower reach by the early 1900s (Snyder 1918; Miller 1969). The species was extirpated from the Mojave River during the twentieth century due to habitat alteration, competition and predation from non-native taxa, and hybridization with arroyo chub (*Gila orcutti*) introduced from coastal streams (Hubbs and Miller 1943; also see Chen et al. 2013). Mohave tui chub were subsequently rediscovered in a spring adjacent to the current site of California State University’s Desert Research Center in Zzyzx, California, near the Mojave River’s terminus into Soda Dry Lake (Miller 1938). Previous or subsequent undocumented translocations resulted in two populations at this site: (1) Mohave Chub Spring (MC Spring), a small (∼3 m in diameter and ∼1.5 m deep) groundwater-fed spring, and (2) a heavily modified spring pool referred to as Lake Tuendae.

*Siphateles bicolor mohavensis* was listed with federal “endangered” status in 1970 (USFWS 1984). Since that time, translocations of Mohave tui chub established populations in several refuge habitats (Miller 1968; Hoover and St. Amant 1983; St. Amant 1983; USFWS 1984; Henkanaththegedara 2012), including one at the Camp Cady State Wildlife Area northeast of Barstow, CA (National Park Service 2004).

Here, we describe the complete mitochondrial genome of *S. b. mohavensis* (accession no. MF972070) sequenced from a fish collected on 11 May 2007 from the Camp Cady Wildlife Area (N 34°56.187´, W 116°36.661´). Tissue from this specimen was deposited into the collections of the Natural History Museum of Los Angeles County (Cat. no. 58482-1, Tissue # T-001269). DNA was isolated from skeletal muscle in 95% ethanol (DNeasy Blood and Tissue Kit, Qiagen, Valencia, CA), and overlapping regions of mtDNA were amplified using GoTaq® Long PCR Master Mix (Promega Corp., Madison, WI) and primers designed to partial sequences of *16S*, *12S,* and *CytB* (AF370043, KM523288, KM435022, KM282481, KM282419, and KM273829). The resulting products were Sanger sequenced by primer walking (MC Lab, South San Francisco, CA) and then assembled (Sequencher v5, Gene Codes Corp., Ann Arbor, MI). The resulting mitogenome was annotated using MitoAnnotator (MitoFish v3.29, Iwasaki et al. 2013).

The complete mitochondrial genome of *S. b. mohavensis* is 16,607 bps in length and encodes 13 protein subunits, 22 tRNAs, and two rRNAs (*12S* and *16S*), as typical of cyprinid mitogenomes. All protein-coding genes were located on the H-strand except *ND6*. The following tRNAs were also identified on the L-strand: *tRNA^Gln^, tRNA^Ala^, tRNA^Asn^, tRNA^Cys^, tRNA^Tyr^*, *tRNA^Ser^, tRNA^Glu^*, and *tRNA^Pro^*. Phylogenetic analysis using complete mitogenomes confirmed that *S. b. mohavensis* belongs to the “western” clade of cyprinids (Colburn and Cavender 1992; Simons and Mayden 1998; Harris 2000; Schönhuth et al. 2012) (Figure 1), which includes the roundtail chub (*Gila robusta*) of the Colorado River Basin, as well as chislemouth (*Acrocheilus alutaceus*) from the Columbia River and Fraser River drainages and hitch (*Lavinia exilicauda*) from central California.

**Figure 1.**
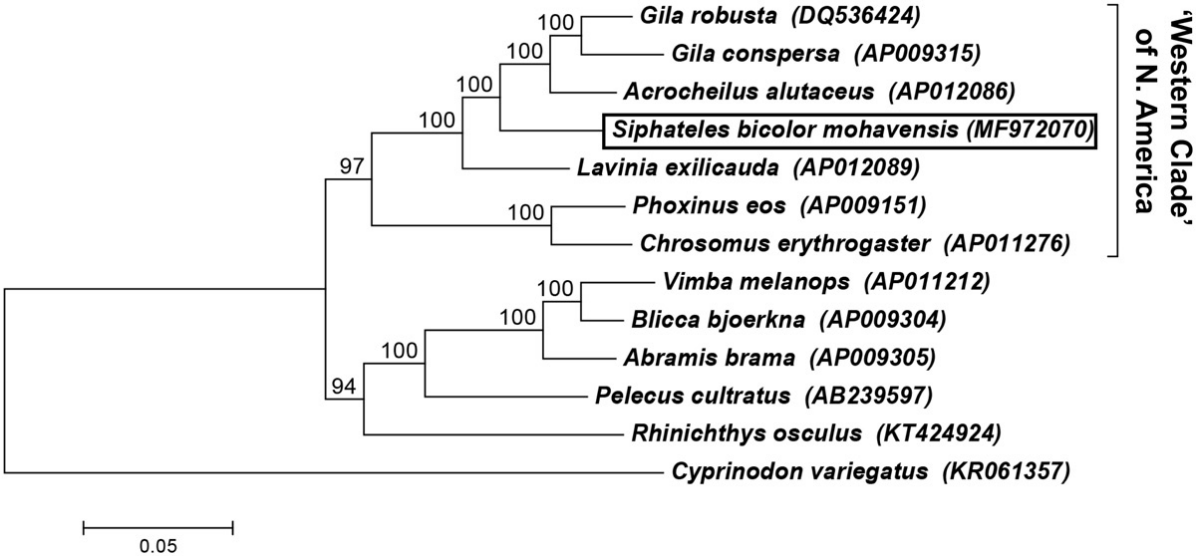
Phylogenomic relationships of *Siphateles bicolor mohavensis* (MF972070) to other fishes inhabiting western North America from family Cyprinidae, order Cypriniformes. Nucleotide sequences from complete mitogenomes were aligned using Clustal X software (Larkin et al. 2007), and a maximum-likelihood phylogenetic tree was generated using a Tamura-Nei Model with all sites in MEGA v7 software (Kumar et al. 2016). Bootstrap values for 1000 replicates are indicated at the nodes. As shown previously in phylogenetic reconstructions using complete *CytB* sequences and partial nuclear DNA sequences (e.g. Schönhuth et al. 2012), *S. b. mohavensis* belongs within the “western North American clade” of Cypriniform genera. The sheepshead minnow (*Cyprinodon variegatus*, order Cyprinodontiformes) was used as an outgroup (Barcelon and Lema 2016). The GenBank accession number for each species’ mitogenome is provided in parentheses.

## References

[CIT0001] Barcelon BR, Lema SC. 2016 Complete mitogenome of the sheepshead minnow *Cyprinodon variegatus* (Cyprinodontiformes: Cyprinodontidae). Mitochondr DNA J Part A. 27:2610–2612. 10.3109/19401736.2015.1041118 26024144

[CIT0002] Blackwater E, Ellsworth EW. 1936 Pleistocene lakes of the Afton Basin, California. Amer J Sci. 31:453–463.

[CIT0003] Buwalda JP. 1914 Pleistocene beds at Manix, in the eastern Mohave Desert region. Univ Calif Dept Geol Bull. 7:443–464.

[CIT0004] Chen Y, Parmenter S, May B. 2013 Genetic characterization and management of the endangered Mohave tui chub. Conser Gen. 14:11–20.

[CIT0005] Colburn MM, Cavender TM. 1992 Interrelationships of North American cyprinid fishes In: Mayden RL, editor. Systematics, historical ecology, and North American freshwater fishes. Stanford (CA): Stanford University Press; p. 328–373.

[CIT0006] Harris PM. 2000 Systematic studies of the genus Siphateles (Ostariophysi: Cyprinidae) from Western North America [dissertation]. Oregon State University; p. 189.

[CIT0007] Henkanaththegedara SM. 2012. Ecological complexity of non-native species impacts in desert aquatic systems [dissertation]. North Dakota State University; p. 174.

[CIT0009] Hoover F, St. Amant JA. 1983 Results of Mohave chub, *Gila bicolor mohavensis*, relocations in California and Nevada. California Fish and Game. 69:54–56.

[CIT0010] Hubbs CL, Miller RR. 1943 Mass hybridization between two genera of cyprinid fishes in the Mohave Desert, California. Papers Michigan Acad Sci Arts Lett. 28:343–378.

[CIT0011] Iwasaki W, Fukunaga T, Isagozawa R, Yamada K, Maeda Y, Satoh TP, Sado T, Mabuchi K, Takeshima H, Miya M, et al 2013 MitoFish and MitoAnnotator: a mitochondrial genome database of fish with an accurate and automatic annotation pipeline. Mol Biol Evol. 30:2531–2540. 23955518 10.1093/molbev/mst141 PMC3808866

[CIT0012] Kumar S, Stecher G, Tamura K. 2016 MEGA7: molecular evolutionary genetics analysis version 7.0 for bigger datasets. Mol Biol Evol. 33:1870–1874. 27004904 10.1093/molbev/msw054 PMC8210823

[CIT0013] Larkin MA, Blackshields G, Brown NP, Chenna R, McGettigan PA, McWilliam H, Valentin F, Wallace IM, Wilm A, Lopez R, et al 2007 Clustal W and Clustal X version 2.0. Bioinformatics. 23:2947–2948. 17846036 10.1093/bioinformatics/btm404

[CIT0014] Miller RR. 1938 Description of an isolated population of the freshwater minnow *Siphateles mohavensis* from the Mohave River basin, California. Pomona Coll J Entomol Zool. 30:65–67.

[CIT0015] Miller RR. 1968 Records of some native freshwater fishes transplanted into various waters of California, Baja California, and Nevada. California Fish Game. 54:170–179.

[CIT0016] Miller RR. 1969 Conservation of fishes in the Death Valley system in California and Nevada. Cal-Neva Wildlife Transact. 1969:107–122.

[CIT0017] National Park Service 2004 Report on a Workshop to Revisit the Mohave Tui Chub Recovery Plan and a Management Action Plan. U.S National Park Service, Mojave National Preserve (15 July 2004); p. 72.

[CIT0018] Schönhuth S, Shiozawa DK, Dowling TE, Mayden RL. 2012 Molecular systematics of western North American cyprinids (Cypriniformes: Cyprinidae). Zootaxa. 3586:281–303.

[CIT0019] Simons AM, Mayden RL. 1998 Phylogenetic relationships of the western North American phoxinins (Actinopterygii: Cyprinidae) as inferred from mitochondrial 12S and 16S ribosomal RNA sequences. Mol Phylog Evol. 9:308–329. 10.1006/mpev.1997.0467 9562988

[CIT0020] Snyder JO. 1918 The fishes of Mohave River, California. Proc U.S. Nat Hist Mus. 54:297–299.

[CIT0021] St. Amant J. 1983 Report on reestablishment of the Mohave chub *Gila mohavensis* (Snyder), an endangered species. Proc Desert Fishes Council. 15:18–19.

[CIT0022] [USFWS] US Fish and Wildlife Service 1984 Recovery plan for the Mohave tui chub, Gila bicolor mohavensis. USFWS, Portland, OR, USA.

